# Photocatalytic Zinc Oxide Nanoparticles in Antibacterial Ultrafiltration Membranes for Biofouling Control

**DOI:** 10.3390/molecules29061274

**Published:** 2024-03-13

**Authors:** Ralfs Vevers, Akshay Kulkarni, Alissa Seifert, Kathrin Pöschel, Kornelia Schlenstedt, Jochen Meier-Haack, Linda Mezule

**Affiliations:** 1 Water Systems and Biotechnology Institute, Riga Technical University, Kipsalas Street 6A, LV-1048 Riga, Latvia; 2 Leibniz Institute of Polymer Research Dresden, Hohe Straße 6, D-01069 Dresden, Germanymhaack@ipfdd.de (J.M.-H.)

**Keywords:** biofouling, drinking water, membranes, photocatalysis, ultrafiltration, zinc oxide

## Abstract

Global water scarcity is a threat that can be alleviated through membrane filtration technologies. However, the widespread adoption of membranes faces significant challenges, primarily due to membrane biofouling. This is the reason why membrane modifications have been under increasing investigation to address the fouling issues. Antibacterial membranes, designed to combat biofouling by eliminating microorganisms, offer a promising solution. Within this study, flat sheet ultrafiltration (UF) membranes with integrated photocatalytic zinc oxide (ZnO) nanoparticles were developed, characterized, and assessed through filtration and fouling tests. The antibacterial properties of the membranes were conducted in static tests using Gram-negative bacteria—*Escherichia coli*—and natural tap water biofilm. The results demonstrated a notable enhancement in membrane surface wettability and fouling resistance. Furthermore, the incorporation of ZnO resulted in substantial photocatalytic antibacterial activity, inactivating over 99.9% of cultivable *E. coli*. The antibacterial activity persisted even in the absence of light. At the same time, the persistence of natural tap water organisms in biofilms of modified membranes necessitates further in-depth research on complex biofilm interactions with such membranes.

## 1. Introduction

Today, over 2 billion people reside in water-stressed regions, a number projected to rise due to climate change and population growth, potentially affecting half of the global population by 2025 and leading to 700 million forced relocations by 2030 [1,2]. Therefore, accessible and comprehensive drinking water treatment must be ensured for all. Ultrafiltration (UF), a long-established and widely employed method in drinking water treatment, effectively removes various contaminants [3], making it a key step even in seawater desalination as an effective pretreatment method [4]. Moreover, small-scale membrane systems offer an innovative approach, utilizing solar energy for filtration [5]. However, UF’s broader adoption is hindered by membrane fouling, causing reduced water flux and separation efficiency [3].

Regular membrane cleaning, involving automated rinsing and chemical washing to remove biofilm and contaminants, is crucial for extending their lifespan [6]. However, traditional cleaning methods faces challenges such as high energy costs. Furthermore, some chemical methods may degrade the membrane, although may not be effective on complete biofilm removal [7]. As membrane cleaning cycles temporarily halt permeate production [5], the reduction in energy consumption during maintenance is crucial for energy conservation and improving clean water output.

Biofouling, caused by bacteria, algae, and other microorganisms, is considered the major type of fouling, contributing to more than 45% of all membrane fouling. This is challenging as microorganisms can regrow even after 99.9% removal [8]. Biofouling involves microorganisms growing on membranes, creating protective biofilm layers of microorganisms and extracellular polymeric substances (EPSs), impervious to disinfection during cleaning [9]. Microorganism-induced fouling reduces water flux and quality, increases transmembrane pressure (TMP) and energy use, and degrades membrane capacity [10].

Various fouling mitigation approaches exist in membrane processes, including feed water pretreatment, nutrient control, hydrodynamic optimization, cleaning, and surface modification methods, including polymer blending, surface grafting, and coating [7]. In the widely adopted membrane fabrication process known as non-solvent-induced phase separation (NIPS) [11], polymer blending has emerged as a practical choice, since it does not require an additional membrane treatment after the casting procedure. However, it can be considered as a surface modification method because it impacts the surface properties through alterations in membrane bulk morphology [12]. Unfortunately, the incorporated material is usually distributed throughout the whole membrane structure, and in such case it should be considered as mostly wasted. However, it is possible to modify just the thin surface layer via membrane co-casting technique, which could lead to membrane manufacturing cost reduction. The modified surface layer of the membrane can be cast on top of the less expensive base layer. For flat-sheet membranes, this is achieved by casting two different casting solutions with a double-blade casting system on a basis of the same principles of traditional casting techniques [13,14].

Most polymeric membranes are made of hydrophobic polymers such as polysulfone (PSU), polyethersulfone (PES), or polyvinylidene fluoride (PVDF) [15], which are prone to fouling [16]. Many fouling mitigation strategies often involve enhancing membrane hydrophilicity with additives such as polyvinylpyrrolidone (PVP) [17]. Additionally, nanoparticles (NPs) have been explored for organic matter degradation during photocatalysis [18]. For example, titanium dioxide (TiO_2_) is a widely studied photocatalyst known for its robust oxidative capabilities, particularly under ultraviolet (UV) irradiation [19]. The conduction and valence band of TiO_2_ are positive, relative to the standard potential of reactive oxygen species (ROS), making it a good photocatalyst [20], and it has already demonstrated effective bactericidal performance in photocatalytic process [21]. However, ZnO NPs, with one-quarter of the cost of TiO_2_ [22], offer the efficient photodegradation of organic pollutants in various pH environments, resulting in lower operational costs and higher efficiency, as well as stability, high photosensitivity, and an optimal bandgap for advanced oxidation processes [23,24]. ZnO NPs are thus promising candidates for water treatment applications. In comparison to TiO_2_, ZnO is considered an even better photocatalyst since the oxidation potential of hydroxyl radicals generated by ZnO is higher [25]. A recent study showed that ZnO indeed had a higher antibacterial activity than TiO_2_ [26].

Usually, it is assumed that the photocatalytic process of ZnO requires UV irradiation. However, the antibacterial activity of ZnO can be triggered by visible light without a UV component. The absorption rate is low, yet it produces enough long-lived excitons, which results in additional ROS [27,28]. ZnO exhibits notable photocatalytic antibacterial properties, although the exact mechanism behind this activity remains not completely understood [29,30]. Thus, the possible mechanisms involved are discussed further in this study.

In addition to microorganism inactivation, ZnO is capable of degrading other organic substances that may be present in the source water [23]; thus, the application of photocatalytic filtration membranes is not limited only to biofouling mitigation, but can rather also be implemented as a novel water treatment method, e.g., to degrade drugs or dyes via photocatalysis [31,32] in combination with membrane filtration.

The practical application of photocatalytic particles in membrane filtration involves the utilization of photocatalytic membrane reactors (PMRs). Photocatalysts can be embedded within membrane structures, and these membranes are subjected to light irradiation during the filtration process [33]. While photocatalytic membranes offer considerable potential for achieving high-performance contaminant degradation in water treatment applications, it is essential to acknowledge that the scalability of these membranes remains limited [34]. In such cases, the useful membrane surface area for irradiation is limited. On the other hand, the antimicrobial properties of ZnO NPs can be attributed to their capacity to harm bacterial cell walls and disrupt DNA replication in non-photocatalytic applications as well [35], making them versatile for both conventional and photocatalytic membrane applications.

Although several ZnO membrane studies have been conducted, antibacterial tests are often limited to basic lab assessments, such as colony counting and disk diffusion assays [36], rather than realistic filtration conditions. While useful as an initial screening, these methods may not reflect the membrane’s antibacterial performance during actual filtration, where hydrodynamic conditions significantly influence the formation of microorganism biofilms on membranes [37]. Moreover, researchers tend to use microorganisms, such as *Escherichia coli* [36,38], which are not common in drinking water [39], and therefore are not part of the biofilm in membrane systems. In many cases, the antifouling properties of the membranes are characterized by non-biological foulants [40]; therefore, the impact of ZnO-doped membranes on microorganisms should be considered as still not fully understood. Lastly, many similar membranes have been synthesized, although dual-layer membranes are often limited to dense membrane preparation, used for desalination and membrane distillation [41] or gas separation [42]. Dual-layer membranes for ultrafiltration, particularly in the context of biofouling prevention, remain largely underexplored. Furthermore, the scarcity of studies examining the antibacterial properties of such membranes underscores the need for further research in this area.

Thus, in this study, dual-layer membranes with an additional modified surface layer, blended with ZnO NPs, were prepared and characterized for their potential to outcompete traditional single-layer membranes. To assess the antimicrobial properties of the membranes, conventional tests, using laboratory monocultures of *E. coli*, were complemented with an assessment of natural water biofilm formation on their surface. The comparison of such test results is crucial to understand whether the conventional microbiological tests using *E. coli* are valid for membranes of drinking water systems.

## 2. Results and Discussion

Multiple membrane types, including untreated reference membranes, PVP and ZnO NP-doped membranes, were successfully prepared and characterized. The preparation and characterization techniques and the composition of the membranes are described in the Materials and Methods section, Part 3.

### 2.1. Membrane Morphology Analysis

**Figure 1:**
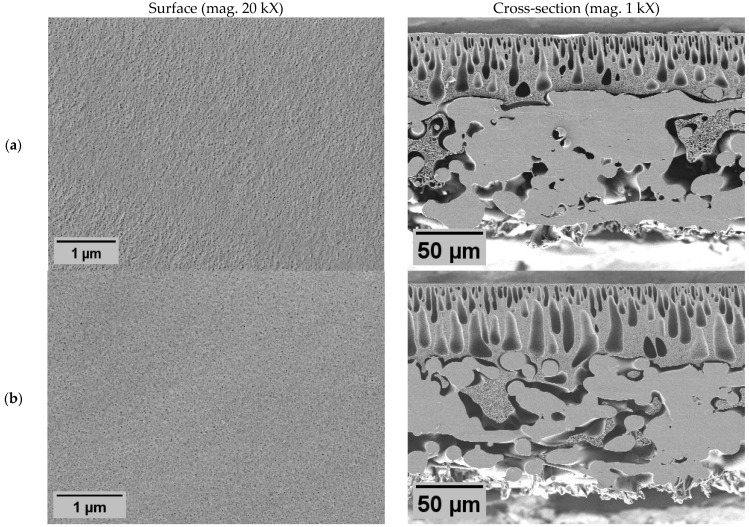
SEM images of prepared non-ZnO membranes: unmodified membrane N1 (**a**); unmodified membrane with PVP N2 (**b**).

**Figure 2:**
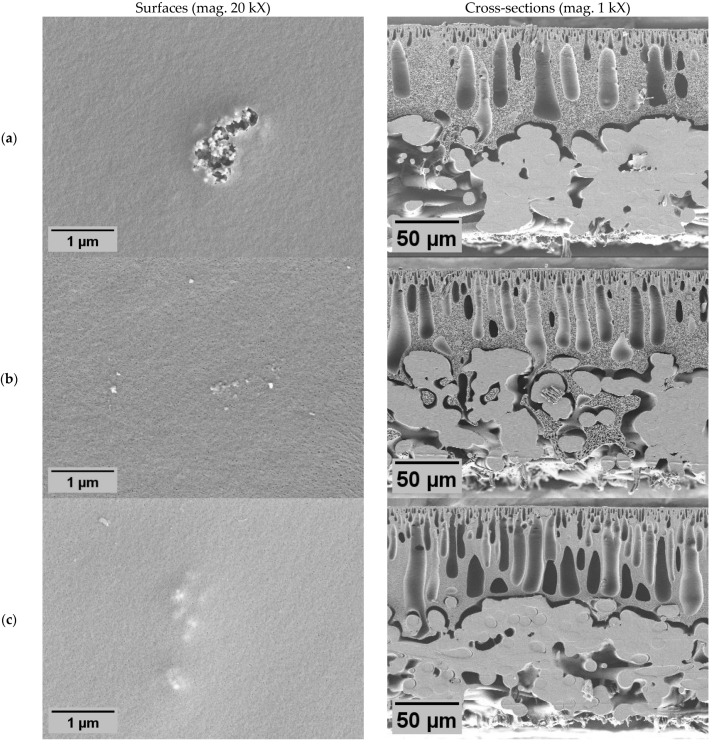
SEM images of prepared ZnO membranes: single-layer ZnO membrane Z1 (**a**); dual-layer ZnO membrane Z2 (**b**); dual-layer ZnO membrane with PVP Z3 (**c**).

Membrane morphology was characterized by scanning electron microscopy (SEM) image analysis (Figure 1 and Figure 2) to demonstrate the impact of different modification approaches: polymer blending with ZnO nanoparticles (NPs), co-casting method, and the impact of PVP.

In all membranes, the classical asymmetric membrane structure was observed, with slight deviations influenced by membrane modifications. The addition of PVP resulted in enlarged macroscopic voids, both in length and diameter, in the cross-section of the membrane with PVP N2 (Figure 1b). At the same time, the surface morphology was not affected since significant differences in pore size or distribution were not observed. From these results, it is expected that the membrane with PVP (N2) would have similar rejection properties as pure PES membrane (N1); similarly, the water flux could be higher due to lower hydraulic resistance, caused by larger internal voids. However, they must be regarded as structural defects, as they have the potential to compromise the mechanical stability of the membrane [43].

The addition of ZnO NPs also resulted in morphological changes in the membranes. Cross-sections revealed large macroscopic voids (Figure 2), indicating an NP role in void formation. In the samples of the dual-layer membrane with PVP (Z3), these voids appeared even longer than in other membranes. Such void formation could be affected by both ZnO NPs and PVP. Irrespective of the surfaces, they were affected by the NPs since the pores of all ZnO membranes appeared to be smaller than in non-ZnO membranes. Although a different method should be used to determine the precise size and distribution of pores, these results indicate that the ZnO membranes probably would have higher particle rejection characteristics. However, the effect on flux was hard to prognose since the size of both pores and internal voids are important. A distinctive feature of membrane Z1 (Figure 2a) was the appearance of large clusters of the NPs on the surface. It is possible that here the agglomerations were so large that the membrane surface appeared broken. However, the crack might also have been a result of sample preparation for SEM during drying of the sample. The same membrane was used in other tests, including filtration.

### 2.2. Distribution of ZnO NPs on Membrane Surface and Bulk

**Figure 3:**
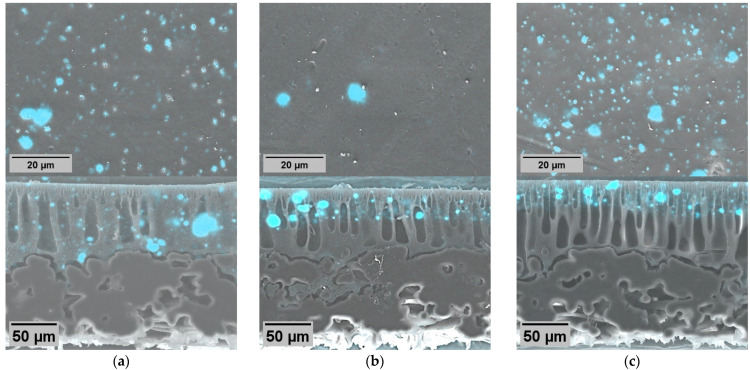
SEM-EDX surface (top line) and cross-sectional (bottom line) elemental maps of ZnO membranes (×1 k; 20 kV): single-layer ZnO membrane Z1 (**a**); dual-layer ZnO membrane Z2 (**b**); dual-layer ZnO membrane with PVP Z3 (**c**). Zn appears in blue.

ZnO NPs exhibit a distinct distribution pattern on the membranes (Figure 3). ZnO NPs were observed to be distributed relatively evenly across the surface. On all membranes, NP agglomerations were observed. At the same time, on the surfaces of the dual-layer membranes without PVP (Figure 3b), the predominant feature is only the presence of large agglomerations of ZnO NPs without any observable fine particles. Here, much larger membrane areas were exposed untreated, which could potentially influence the antimicrobial activity.

Regarding the cross-sections, the added NPs were observed as fine particles or larger particle agglomerates. Such agglomerates were also observed in previous studies [44]. Similar effects can be seen in the cross-section of the dual-layer membrane with PVP. In the case of the dual-layer membranes (Figure 3b,c), it is worth emphasizing that there is a risk of delamination between the two membrane layers [45], especially if the surface layer of the polymer becomes more hydrophilic than the base layer [46]. However, in this study, both layers of the dual-layer membranes were strongly bonded together. The seamless boundary between the polymer layers suggests that during membrane casting, the base layer had not yet initiated precipitation before the surface layer was cast on top of it. Otherwise, this phenomenon might be influenced by ambient humidity levels, like in the vapor-induced phase separation process [47].

In both dual-layer membranes (Z2 and Z3), the NPs were primarily observed in proximity to the surface with a clear boundary layer, which was expected. The thickness of the surface layer, containing the NPs, varied from approximately 30 to 50 µm within a membrane (Figure S1). The observed number of fine particles in membrane Z2 seemed slightly lower than in membrane with PVP (Z3), although the concentration in both casting solutions was the same. Additionally, the particles in membrane Z3 were distributed closer to the surface.

**Figure 4:**
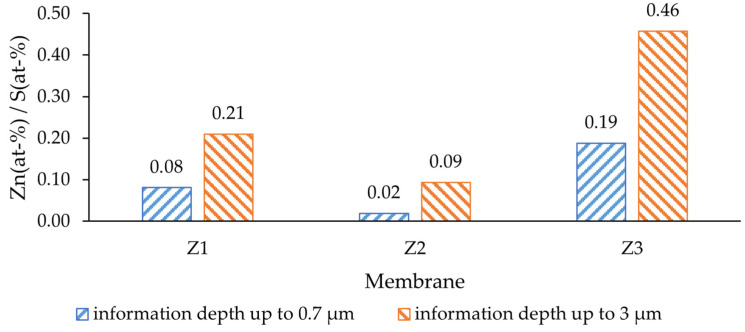
Surface composition of membranes at different excitation voltages: 6 kV—information depth of up to 0.7 µm; 20 kV—information depth of up to 3 µm. Z1—single-layer ZnO membrane; Z2—dual-layer ZnO membrane; Z3—dual-layer ZnO membrane with PVP.

The modified casting solution contained 20 wt-% PES (232 g/mol; MW of repeating unit) and 5 wt-% ZnO (81.4 g/mol), the concentration of each was 86.2 mmol (PES) and 61.4 mmol (ZnO). This resulted in a Zn/S atom ratio of 0.71, which was expected to be found on the surface of the modified membranes. Within a depth of up to 0.7 µm from the membrane surface, the amount of ZnO was significantly lower than expected (Figure 4). The deeper layers, up to 3 µm from the surface, showed a higher Zn presence, yet still not reaching the nominal value (0.71). This difference indicates that the NPs were mostly covered by a film of PES and would not be in direct contact with the feed water. The lowest ratio was determined for the dual-layer membrane without PVP (Z2), where in a depth of up to 0.7 µm the Zn/S ratio was only 0.02. On the other hand, the highest Zn/S ratio was observed for the dual-layer membrane with PVP (Z3); therefore, the antibacterial activity of this membrane was expected to be higher than of others. Such a difference could be explained by the effect of PVP, as it alters the pore-forming process [48] and could potentially bring the solid particles closer to the surface during precipitation.

NPs could probably contribute to reducing pore blockage with different foulants that may potentially be degraded by ZnO. However, in cases of biofouling, the NPs inside the membrane should be considered as wasted, since the microorganisms are larger than the pores, and therefore could only promote surface fouling.

### 2.3. Membrane Surface Wettability

**Figure 5:**
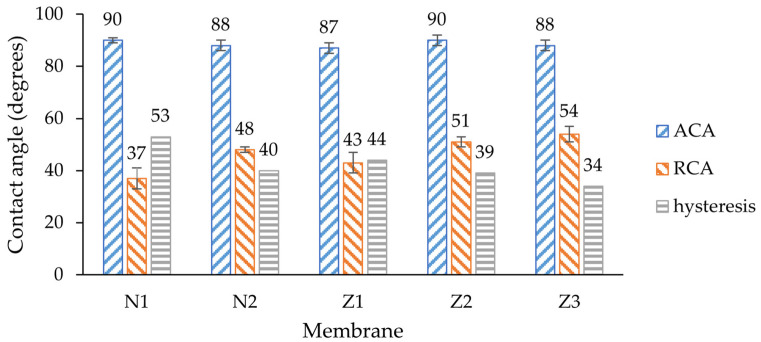
Dynamic contact angle measurements: advancing contact angle (ACA), receding contact angle (RCA) and hysteresis. N1—unmodified membrane; N2—unmodified membrane with PVP; Z1—single-layer ZnO membrane; Z2—dual-layer ZnO membrane; Z3—dual-layer ZnO membrane with PVP.

To characterize the wettability, dynamic contact angle measurements were employed (Figure 5), a less common approach compared with the widely discussed static contact angle determination technique employed in most articles. Measuring the static contact angle alone may not provide a comprehensive understanding of a surface’s wetting behavior. Here, the hysteresis, which is the difference between advancing and receding contact angles, provides insights into surface irregularities and the reversibility of wetting, both of which are important for assessing hydrophilicity [49].

The average advancing contact angles (ACAs) of all membranes were around 89°, indicating the hydrophobic nature of the membranes. Since the ACA shows the angle of the droplet being pushed to the surface, the snap-in force affects the shape of the droplet [50]. Here, any modification including only PVP or ZnO incorporation led to slight ACA improvement by few degrees. The exception is the dual-layer membrane without PVP, where the ACA did not change. This could be related to the fact that the number of ZnO NPs on the surface was the lowest amongst all other membranes.

The receding contact angles (RCAs) differed for each membrane, and any modification led to a significant RCA increase. A lower RCA value implies that the liquid is more likely to be retained and spread across the surface, indicating hydrophilic behavior. Such behavior alone would suggest that membranes became more hydrophobic, but here it needs to be evaluated in context of ACA; therefore, hysteresis is important. The lowest RCA was observed on the non-ZnO membrane without PVP (37 ± 4°); therefore, the hysteresis of this membrane was the highest (53°). By adding PVP, the RCA increased by 11°, which means that the membrane surface had become more homogenous, and the liquid was easier to retract due to a lower pull-off force [50]. These results align with other studies where PVP increased the hydrophilicity of the membranes [51]. The RCA of a single-layer ZnO membrane was 6° higher than non-ZnO membrane, which also means that surface homogeneity improved due to the addition of ZnO NPs.

The RCA of the dual-layer membrane increased by 8° compared with the single-layer membrane, which could mean that the surfaces or internal composition of those ZnO membranes differ. The same effect of PVP was observed in ZnO membrane samples where the RCA of the membrane with PVP increased by 3° in comparison to a similar sample without PVP.

The dual-layer membrane with PVP showed that surface homogeneity can be improved by combining both ZnO NP and PVP incorporation in membrane, as the hysteresis of this membrane was the lowest amongst all other membranes. Therefore, higher fouling resistance and antimicrobial activity was expected for this type of membrane. The results of ZnO membranes overall align with other studies where the hydrophilic properties of similar membranes increased [40].

### 2.4. Permeability and Fouling Characteristics

**Figure 6:**
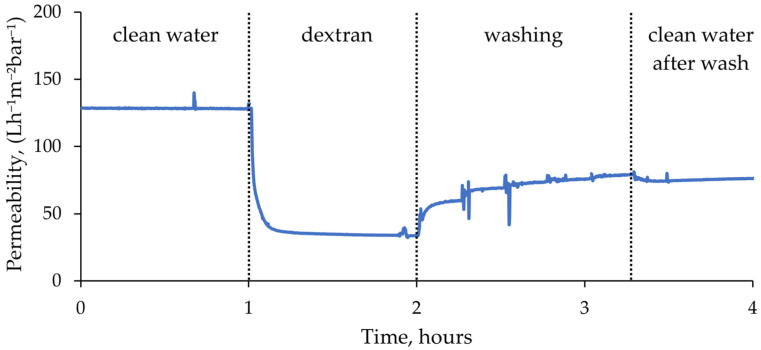
Filtration curve example of unmodified membrane N1 at constant TMP = 1 bar.

The filtration tests were performed using an automatic membrane testing device, which enabled automatic switching between feed solutions and data logging. This resulted in a continuous uninterrupted filtration curve for each membrane (see example in Figure 6).

In the filtration tests, a stable permeate flux was predominantly achieved within the initial 4 h of membrane compaction. Subsequently, the flux remained constant, and thus, the average flux value during the final hour of compaction was taken as the clean water permeability of the membranes. Once the feed switched to dextran solution, the flux dropped dramatically, which indicated rapid membrane fouling. However, after a few minutes, a relatively stable permeate flow was established. The membranes fouled until a level after which foulant could not accumulate anymore. During washing cycles, most of the flux was recovered at the first step of 15 min, and at the fifth and final step the changes in flux were minimal. Lastly, clean de-ionized (DI) water filtration curve after washing remained constant.

**Figure 7:**
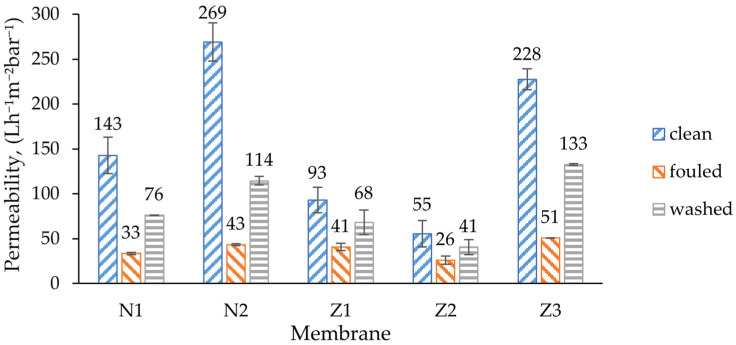
Clean water permeability and permeability during membrane fouling and washing. N1—unmodified membrane; N2—unmodified membrane with PVP; Z1—single-layer ZnO membrane; Z2—dual-layer ZnO membrane; Z3—dual-layer ZnO membrane with PVP.

In the filtration curves, especially during washing, multiple spikes were observed. This was mostly caused by measuring errors during switching between the tanks. For average permeability calculations (Figure 7), such faulty values were excluded from the data. Average filtration curves of all membranes are presented in Figure S2.

The initial permeability of unmodified membrane was 143 L/(h·m^2^·bar), which almost doubled to 269 L/(h·m^2^·bar) when PVP was used in a non-ZnO membrane. This can be explained by the well-known ability of PVP to increase the flux, as was also found in a study by Soundarrajan et al. [52]. The same effect of PVP could be observed in modified dual-layer ZnO membranes where the addition of PVP more than quadrupled the initial clean water flux of the non-PVP membrane (Z2) from 55 to 228 L/(h·m^2^·bar).

Comparison of ZnO membranes with non-ZnO membranes showed that clean water flux decreases once ZnO NPs are implemented. The initial flux of the modified single-layer membrane was 35% lower than that of a comparable unmodified membrane. The decrease can be explained with the decrease in pore size of the ZnO-doped membrane, but the results contradict with previous studies where the flux increased by adding more ZnO NPs [53]. On the other hand, Ahmad et al. found that there is a limit of ZnO NP content after which porosity decreases and also affects the flux [40]. Similar results were observed by Alsalhy et al. for PVC membranes [54]. During membrane fouling, the flux decreased to a certain amount, which was similar for all membrane types.

**Figure 8:**
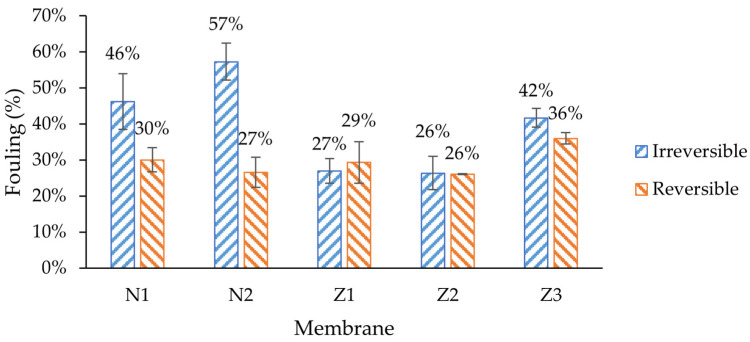
Irreversible and reversible fouling characteristics in dextran filtration tests. N1—unmodified membrane; N2—unmodified membrane with PVP; Z1—single-layer ZnO membrane; Z2—dual-layer ZnO membrane; Z3—dual-layer ZnO membrane with PVP.

**Figure 9:**
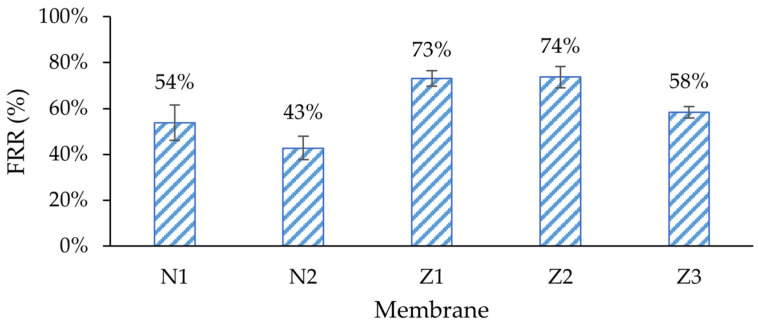
Flux recovery rate (FRR) after membrane washing cycle with de-ionized water. N1—unmodified membrane; N2—unmodified membrane with PVP; Z1—single-layer ZnO membrane; Z2—dual-layer ZnO membrane; Z3—dual-layer ZnO membrane with PVP.

Amongst all membranes, the non-ZnO membranes had higher irreversible fouling, especially in membranes with PVP, where 57% of its original pure water flux was irreversibly lost during fouling (Figure 8). The flux recovery rate (FRR) of the membrane with PVP (N2) was the lowest (Figure 9). The same behavior could be observed when modified ZnO membranes without PVP (Z1, Z2) were compared with the membrane with PVP (Z3). Traditionally, PVP is employed as a membrane additive to enhance fouling resistance and flux stability [55]. However, in this case, it appears to diminish the membrane’s capacity to recover its initial flux. In the context of pure water flux data, it can be assumed that the high flux of PVP membranes comes with a price of much higher fouling. This can be explained with higher pore blocking, caused by enlarged pores at the surface. In such cases, the lower-molecular-weight dextran could have entered the pores more deeply; therefore, they could not be rinsed easily.

It was expected that ZnO membranes would have improved antifouling properties by enhancing their surface properties, e.g., hydrophilicity. Indeed, the lowest irreversible fouling was observed in modified single- and dual-layer ZnO membranes, 27% and 26%, respectively, despite obvious differences in surface composition, as shown in the morphology analysis sections above. This resulted in the highest FRR (73% and 74%) amongst other membranes (Figure 9). It seemed that the added ZnO NPs improved the fouling resistance and enhanced membrane performance, which was consistent with findings in similar studies [53]. The reversible fouling of all membranes was similar, with a notable exception for the membrane with PVP and ZnO (Z3), where it was the highest (36%). On the other hand, the FRR of membrane Z3 was lower than that of the other ZnO membranes, and was close to that of the unmodified membrane N1.

**Figure 10:**
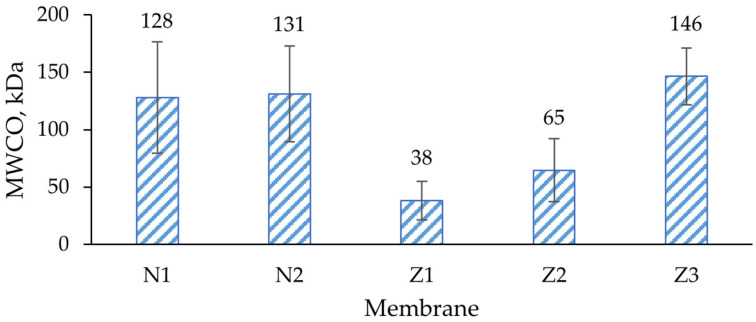
Dextran molecular weight cut-off (MWCO) of membranes. N1—unmodified membrane; N2—unmodified membrane with PVP; Z1—single-layer ZnO membrane; Z2—dual-layer ZnO membrane; Z3—dual-layer ZnO membrane with PVP.

This reduction in fouling was probably not completely related to ZnO interactions with the foulant; instead, it can be explained with different type of fouling. The MWCO values of membranes Z1 and Z2 were lower than in other membranes (Figure 10), indicating a higher rejection of large molecules. In SEM surface analysis, it was found that the pore size of ZnO membranes appeared smaller than of pure PES (Figure 1 and Figure 2). The pore size was calculated from the GPC curves, which also partially confirmed it (see further). In such cases, the fouling probably did not occur internally in membranes Z1 and Z2, but mostly on the surface, since most of the dextran mixture mass consisted of larger-molecular-weight molecules than the MWCO of Z1 and Z2. Therefore, the membranes could be washed with clean DI water more easily, which resulted in higher FRR. The changes in wettability in the modified membranes (Figure 5) likely did not affect the fouling resistance much.

### 2.5. Separation Properties

SEC/GPC measurement results and molecular weight cut-off (MWCO) determination are presented in Figure S3. Both non-ZnO membranes had similar dextran MWCO values—128 and 131 kDa, respectively—which can be characterized as loose UF membranes (Figure 10). Despite significant increases in the flux of non-ZnO membranes with added PVP, the rejection properties did not change. This means that PVP can be implemented in flux regulation while maintaining the same rejection properties.

A significant decrease in MWCO was observed in both ZnO-modified single- and dual-layer membranes. This indicated an enhanced rejection of smaller dextran molecules. The observed drastic reduction in MWCO of the ZnO modified membranes here could be attributed to the narrower pore size distribution resulting from the interaction between ZnO and the membrane matrix, as it was observed in previous studies [44]. However, Chung et al. observed increases in porosity and pore size in their ZnO membranes [56]. Although it was found that the surface of membrane Z1 seemed to be broken (Figure 2a), here it seems that such morphology did not affect the filtration properties of the membrane since the MWCO was the lowest among all samples tested.

Although pure water flux of the dual-layer ZnO membrane was lower than that of a single-layer ZnO membrane, the MWCO was almost twofold higher. This could be explained by the lower pore distribution density, as well as the increased pore size of this dual-layer membrane. Lastly, the addition of PVP to a dual-layer ZnO membrane resulted in an MWCO increase, reaching 146 kDa, which was the highest amongst all membranes. This means that the effect of PVP is not the same for all types of membranes, and in ZnO-doped membranes it does not maintain the same rejection properties; therefore, it cannot be used for flux regulation.

### 2.6. Pore Size and Pore Distribution Analysis

**Figure 11:**
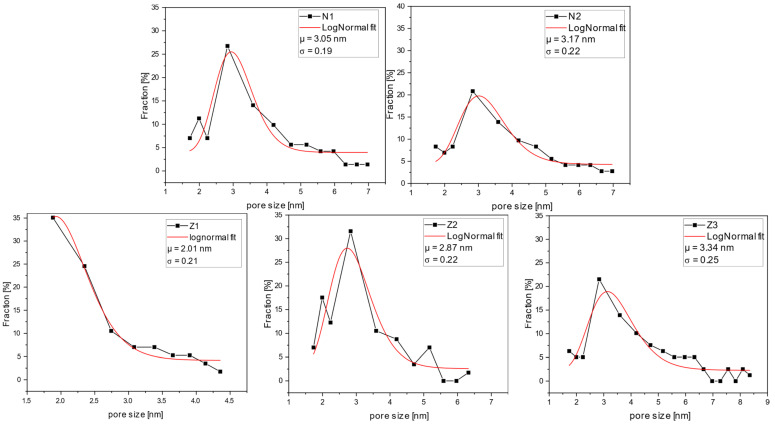
Permeate sample GPC analysis: pore size and pore distribution from dextran rejection. N1—unmodified membrane; N2—unmodified membrane with PVP; Z1—single-layer ZnO membrane; Z2—dual-layer ZnO membrane; Z3—dual-layer ZnO membrane with PVP.

GPC curves illustrate the percent rejection for the specific molecular weights of dextran (Figure 11). The range of molecular weights was selected, and the data points for the percentage fraction were obtained by calculating the difference between the respective rejection values of higher and lower molecular weights of the selected range. The average molecular weight of the range was calculated by averaging higher and lower molecular weights of the range and further utilized for size evaluation.

All the membranes, except for sample Z1, had a relatively narrow pore size distribution, with an average of around 3 nm. A lower average pore diameter of 2 nm was found for membrane Z1. However, it is noticeable that by adding PVP to the casting solution, both average pore sizes increased and the pore size distributions became broader. Furthermore, the dual-layer membranes, Z2 and Z3, had a larger average pore size than the single-layer membrane (Z1). In sample Z3, this effect was further enhanced by the addition of PVP. This aligns with the results of the SEM and MWCO analyses. However, here, the impact of PVP on pore size increase can be seen more clearly.

### 2.7. Antimicrobial Properties

In static conditions, the unmodified membrane without PVP showed a bacterial growth of 4.6 ± 0.1 log. This type of membrane was tested together with ZnO membranes, and was also irradiated. In this case, the non-ZnO membrane with PVP (N2) was not tested alone since the PVP was not considered to have antibacterial properties. Moreover, a strong antibacterial effect was observed on all ZnO membrane samples (Table 1 and Figure S4). All types of ZnO membranes showed similar efficacy in cultivable *E. coli* inactivation, despite differences in the distribution of ZnO NPs on the surface. After 24 h of irradiation, the reduction in cultivable *E. coli* reached the detection limit of >3.2 log, indicating a >99.9% reduction in cultivable *E. coli*. The antibacterial effect was also observed in ZnO membrane samples without any irradiation although the reduction was lower. The average reductions reached 2.6 ± 0.1 log, 2.5 ± 0.4 log, and 2.0 ± 0.3 log on single- and double-layer membranes with and without PVP, respectively. Thus, the inactivation of >99% of bacteria for all membranes in the dark was obtained. These results correspond to similar tests where more than 3 log reductions were observed on ZnO surface coatings [57].

The dual-layer ZnO membrane with PVP had the highest concentration of NPs on the surface; thus, it was expected that antibacterial activity would be the highest. In practice, the *E. coli* inactivation rate in the dark was the lowest among other membranes. Moreover, the dual-layer membrane without PVP showed higher antibacterial activity, which did not correlate with the amount of ZnO on the surface. This could mean that there is a threshold of ZnO concentration after which the activity does not increase by more than 2 log. Therefore, the impact of added ZnO should be evaluated in further studies.

In samples where membrane pieces were placed on agar, no colonies were found under irradiated membrane pieces (Figure S5), which means that either these membranes maintained higher antibacterial properties after irradiation and were more active during incubation on agar plate, or the whole suspension had become inactivated for each irradiated ZnO membrane. On the other hand, some colonies were observed under non-irradiated membrane samples, which proves the photocatalytic antibacterial activity of ZnO membranes.

The possible mechanisms of antibacterial activity include the release of ROS, which would cause an oxidative stress in bacteria by damaging the DNA. This can be induced by photocatalytic activity during which H_2_O_2_ is released. Other explanations include a release of Zn^2+^ ions, damaging the cell membrane and penetrating the internals, and direct contact with the bacterial cell membrane by a ZnO particle [58,59]. This can explain the antibacterial activity during illumination and in the dark; during photocatalysis, the ROS were produced additionally to other mechanisms. However, in the absence of any light, the bacteria were still impacted by a release of Zn^2+^ ions near the surface. The impact of direct contact here is considered minimal, since in EDX analysis it was determined that there were minimal ZnO NPs on the surface and they were slightly submerged in the polymer. This raises a question regarding the possible movement of released zinc ions and ROS away from the surface under actual filtration conditions.

### 2.8. Tap Water Biofilm Growth on Membrane Surfaces

In both non-ZnO and ZnO membrane samples, bacterial cells were observed adhering to the membrane surface. The persistence of these cells following sample rinsing suggests initial attachment and potential colonization, as they resisted detachment from the membrane during the sample rinsing. Furthermore, the examination of metabolically active—CTC-positive—cells showed that all samples contained a significant population of metabolically active cells with an operating electron transport chain (ETC), indicative of their viability.

**Figure 12:**
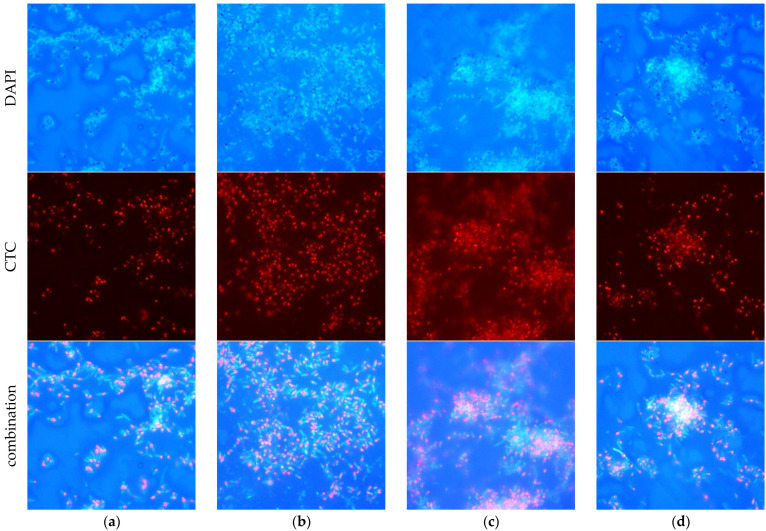
Multi-channel fluorescence microscopy images of tap water microorganism biofilm on membrane surfaces, stained with 4′,6-diamidino-2-phenylindole (DAPI) and 5-cyano-2,3-ditolyltetrazolium chloride (CTC): unmodified membrane as a reference (**a**); single-layer ZnO membrane (**b**); dual-layer ZnO membrane (**c**); dual-layer ZnO membrane with PVP (**d**).

However, intensive background luminescence was caused by DAPI stain binding to the polymer in the membrane. Therefore, evaluation and good-quality image acquisition were difficult. Eventually, images of tap water biofilm on membrane surfaces were prepared (Figure 12), although the image acquisition method could be improved.

In the context of the static test results, it appears that bacteria in contact with ZnO membranes lose their ability to form colonies, but remain metabolically active. This behavior is reminiscent of the active but non-culturable (ABNC) state, also called the viable but non-culturable (VBNC) state observed in previous studies [60,61]. It suggests the need to investigate whether bacteria can regain their ability to multiply, since it was observed that bacteria formed microcolonies on the ZnO-modified membrane surface (Figure 12b–d), represented as large clusters of bacterial cells. Thus, the bacteria had the ability to interact with other species, which could contribute to biofilm formation. The presence of biofilms on all these samples indicates that ZnO did not inhibit the growth of bacteria, nor the enhanced surface of the membrane with ZnO, and PVP was able to prevent the attachment of bacteria. The question at hand is whether conducting static cultivation tests is a meaningful approach for evaluating antibacterial properties.

### 2.9. Comparison of Microbiological Tests Results to Similar Studies

Many experiments with similar membranes have been conducted by different authors; most of them have used *E. coli* for their antibacterial tests (Table 2). This work shows that it is possible to achieve an even higher antibacterial activity against *E. coli* by manufacturing membranes through a simpler technique. However, despite having the highest antibacterial activity amongst the mentioned examples, the membranes in this study did not inhibit the biofilm formation that occurred in conventional tap water. Therefore, the choice of the test organism plays a significant role in the evaluation of filtration membranes.

## 3. Materials and Methods

### 3.1. Preparation of Membranes

Flat sheet membranes were prepared using non-solvent-induced phase separation (NIPS) by the immersion precipitation technique. A PES (Ultrason^®^ E 6020 P, BASF, Ludwigshafen, Germany) polymer was dissolved in NMP (Merck, Darmstadt, Germany) to prepare casting solutions with a consistent concentration of 20 wt-% PES. These solutions were supplemented with 5 wt-% ZnO NPs (GetNanoMaterials, Saint-Cannat, France) and 1 wt-% PVP K25 (Fluka Chemie, Buchs, Switzerland). After thorough mixing at 400 RPM overnight in a shaker (KS 130 basic, IKA, Staufen, Germany), the solutions achieved homogeneity. More details about the prepared membranes and their types are provided in Table 3.

Membranes were cast using a calibrated blade onto a polyester non-woven support sheet (novatexx 2484; Freudenberg, Weinheim, Germany). Two membrane types were created: non-ZnO single-layer membranes, used as reference and for PVP impact evaluation; and ZnO membranes, in both traditional single layers and dual layers, with and without PVP configurations, using the co-casting technique described by Fu et al. [13]. In dual-layer membranes, the base layer was pure PES, and the surface layer was PES plus additives (ZnO and PVP). The composition of casting solutions is summarized in Table 3. Polymer precipitation was performed in de-ionized (DI) water immediately after casting.

### 3.2. Characteristics of ZnO Nanoparticles

The obtained ZnO nanoparticles were in nanopowder form, with a nominal size of 20 nm. These NPs were non-porous or macro-porous in nature and showed type 3 adsorption isotherms with H3 desorption hysteresis loops, which indicated non-rigid aggregates [66]. The measured surface area of particles was 17 m^2^/g (Figure S6). SEM images of the NPs are presented in Figure S7.

### 3.3. Surface and Cross-Section Morphology Characterization

Membrane morphologies were characterized by scanning electron microscopy (SEM) image analysis. The distribution of ZnO particles was analyzed using energy-dispersive X-ray spectroscopy (EDX).

Cross-sections of the membranes were prepared utilizing an ultramicrotome UC7 (Leica, Wetzlar, Germany) at −170 °C. Prior to SEM measurements, all samples were coated with a layer of 3nm Pt. SEM images were acquired using a Gemini Ultra plus (Zeiss, Oberkochen, Germany). The applied excitation voltage was 3 kV. For EDX measurements, excitation voltages of 6 kV and 20 kV were applied to obtain information depths of up to 0.7 µm and 3 µm, respectively.

### 3.4. Contact Angle Measurements

Membrane hydrophilicity properties were characterized through dynamic contact angle measurements. The dynamic contact angles were determined through a special form of Axisymmetric Drop Shape Analysis (ADSA-P): the “captive bubble” method. Membranes were held in a frame and immersed in water. An air bubble was created under the membrane, enlarged, and then reduced again. The profiles were extracted from the experimentally determined bubble images and adapted to theoretical profiles by varying different parameters of the Laplace equation, for which special evaluation software (ADSA, version 3.0., 2005) was used. The software was developed by supervision of Dr. A.W. Neumann in the Laboratory of Applied Surface Thermodynamics, Department of Mechanical and Industrial Engineering at the University of Toronto. The device for measurements was self-made.

In this case, advancing contact angles (ACAs) and receding contact angles (RCAs) were measured. The ACA measures a general surface’s tendency to be wetted by a liquid, indicating its hydrophilic or hydrophobic nature. In contrast, the RCA is a measure of relative hydrophilicity [67], which assesses how effectively the surface either retains a liquid or causes it to bead up and retract, reflecting its repellent or retentive characteristics.

### 3.5. Filtration Performance, Fouling Analysis, and Rejection Properties

Filtration tests were performed on an LSta10-SPS automatic membrane testing device (SIMA-tec, Schwalmtal, Germany). Membrane samples were cut and placed in a homemade plexiglass crossflow filtration cell with a membrane area of 114 cm^2^. Filtration was performed under constant TMP of 1 bar. The crossflow rate was set at a constant 60 L/h. The concentrate and permeate were recirculated back into the feed tank.

Initially, membranes were compacted by filtering DI water overnight until stable permeate flux was achieved. For fouling characterization, filtration tests were performed for 1 h by the filtration of an aqueous dextran solution (1 g/L) containing equal amounts of dextran with different molecular weights (2.5, 6, 10, 20, 40, 70, 100, 250, and 500 kDa; Carl Roth, Karlsruhe, Germany). After dextran filtration, the membranes were rinsed by filtering clean DI water 5 times for 15 min. During rinsing, the TMP setting was 0 bar. Between rinsing cycles, feed water was replaced and the tank was briefly rinsed manually. Lastly, clean DI water was filtered again for 1 h.

For fouling analysis, the permeability was also calculated for dextran filtration and second clean water filtration after wash. The flux recovery rate (FRR, %) was measured by comparing permeability values of clean ($L_{p.clean}$) and washed ($L_{p.washed}$) membranes:

(1) $$FRR\;\left(\%\right)=\frac{L_{p.washed}}{L_{p.clean}}\cdot 100$$

Total fouling was calculated to characterize the percentage of initial flux that was decreased during fouling tests:

(2) $$F_{tot}\left(\%\right)=1-\frac{L_{p.fouled}}{L_{p.clean}}\cdot 100$$

Irreversible fouling was part of total fouling that could not be recovered after membrane rinsing:

(3) $$F_{ir}\;\left(\%\right)=1-\frac{L_{p.washed}}{L_{p.clean}}\cdot 100$$

Reversible fouling, on the other hand, here represents the amount of flux lost during fouling, but subsequently recovered after membrane rinsing. It was calculated as the difference between total fouling (%) and irreversible fouling (%):

(4) $$F_{rev}\;\left(\%\right)=F_{tot}-F_{ir}$$

The molecular weight cut-off of membranes samples was determined by the filtration of dextran solution samples containing dextran in the molecular weight range from 2.5 kDa to 500 kDa. The molecular weights (MWs) and molecular weight distribution in the feed and permeate samples were obtained via GPC measurements. These were conducted on a Knauer GPC equipped with two PL aquagel OH-MIXED-H (8 µm) columns, an RI detector K-2301 (Knauer, Berlin, GermanyAn aqueous solution containing 0.01 M NaH_2_PO_4_ (pH = 7) and 0.2 M NaNO_3_, as well as 0.02% NaN_3_, was used as an eluent and at a flow rate of 1 mL min^−1^ (25 °C) maintained by a 1260 isocratic pump (Agilent Technologies, Santa Clara, CA, USA). Polysaccharide standard samples (PL laboratories, Richmond, BC, Canada) served as standards for molecular weight calibration.

(5) $$R\left(\%\right)=1-\frac{permeate\;response\;signal,\;\mathrm{mV}}{feed\;response\;signal,\;\mathrm{mV}}\cdot 100$$

The dextran rejection (R,%) was calculated at each MW by comparing the respective response signals without calculation of the actual concentration. The MWCO is given as an average MW value at a 90% dextran rejection.

### 3.6. Pore Size and Pore Distribution Analysis

The mean pore sizes and pore size distributions were roughly estimated from the GPC curves (permeate) of dextran filtration, under the assumption that the separation was based on a pure sieving effect and that the dextran molecules are present as hydrated spheres. The hydrodynamic radii ($r_{hydrated}$, Å) were converted from the molecular weights according to Venturoli and Rippe [68]:

(6) $$r_{hydrated}=0.33\cdot MW^{0.463}$$

where MW is the molecular weight in Da.

The average pore sizes and the pore size distributions were calculated by fitting the measured values using the LogNormal distribution function:

(7) $$y=y_{0}+\frac{A}{\sqrt{2\pi }\;\sigma x}e^{\frac{-{\left[ln\frac{x}{\mu }\right]}^{2}}{2\sigma^{2}}}$$

where A is the area, $y_{0}$ is the *y*-axis offset, x is the x-value, μ is the weighted average pore size (nm), and σ the standard deviation.

### 3.7. Antibacterial Tests of Membrane Surfaces

A static test was used for the evaluation of membrane surface antibacterial properties. Gram-negative *Escherichia coli* ATTC^®^25922 was inoculated into sterile tryptone soya broth (TSB, CM0129, Oxoid, Basingstoke, UK) and cultivated overnight in a shaker at 150 rpm and 37 °C (Biosan, Riga, Latvia). The next day, the suspension was centrifugated at 6000 rpm for 2 min (Minispin, Eppendorf, Hamburg, Germany) and the culture medium was replaced with sterile 0.1% bacteriological peptone (Biolife Italiana, Milano, Italy). The washing cycle was repeated 3 times in total, after which a nutrient-free *E. coli* suspension (~10^9^ cells/mL) was obtained. To determine the exact cell concentration, 0.005 mL of suspension was filtrated through a sterile 25 mm diameter 0.2 µm pore size filter (Polycarbonate Track-Etch Membrane, Sartorius, Göttingen, Germany). Cells were fixed with 3–4% formaldehyde for 10 min, rinsed with sterile DI water, and stained with 10 µg/mL DAPI (4′6-diamidino-2-phenylindole, Merck, Germany) for 10 min. The cell concentration was determined with an epi-fluorescence microscope (Ex: 340/380; Em: >425; Axioscope 5, Carl Zeiss, Oberkochen, Germany) by counting 20 random fields of view. The stock suspension was prepared by diluting the clean *E. coli* suspension in sterile diluted TSB in DI water (1:10) to a concentration of 10^4^ cells/mL.

Each membrane sample was cut into 2 × 2 cm squares, disinfected in ethanol, and put in separate sterile dishes. Subsequently, 1 mL of stock suspension was put onto the membrane surface and left for 24 h at room temperature. Membranes were illuminated with a commercial lamp (50 W, 3000 K, 5250 lm, Ledvance, Garching bei München, Germany) which was fixed 30 cm above the samples. After incubation, decimal dilutions of samples were prepared in sterile 0.1 % peptone water and inoculated on tryptone bile x-glucuronide (TBX, Oxoid, Basingstoke, UK) agar plates. Antibacterial properties were estimated as reductions in the number of colony forming units (CFUs) after 24 h against the initial cell concentration in stock suspension:

(8) $${\mathrm{logreduction}=\log}_{10}\left(\frac{\mathrm{initial}\;\mathrm{CFU}}{\mathrm{final}\;\mathrm{CFU}}\right)$$

Finally, all membrane samples were also placed on a TBX agar plate to evaluate the growth of colonies in contact with the membrane surface. ZnO should exert antibacterial effects without photocatalysis, as discussed in the Introduction; therefore, the same test was repeated in the dark without irradiation to evaluate the differences in the results.

### 3.8. Light Source Characteristics

The LED lamp spectra were taken using a CCD spectrometer (Ossila, Sheffield, UK) with an integration time of 10 ms. Additionally, a shortpass filter was installed and the integration time was increased to 40 ms to better distinguish the high-energy wavelength range.

**Figure 13:**
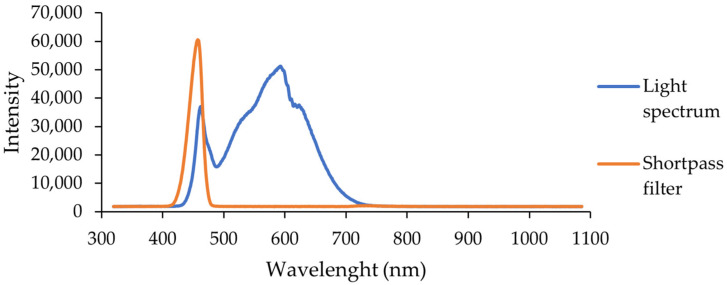
Spectrum of the LED lamp used for sample illumination.

The lamp used for sample illumination was a visible light lamp. It emitted only visible light wavelengths, without any ultraviolet component below 400 nm (Figure 13).

### 3.9. Evaluation of Natural Microbial Biofilm Formation

*E. coli* was used in the antibacterial tests, which did not represent natural water microbiota [39]; therefore, tests with natural biofilm grown on the membranes were performed. Membrane pieces of 5 × 5 mm in size were immersed in a sterile disposable plastic cup, and filled with 50 mL of unfiltered tap water from Riga, Latvia (Table 4), containing natural tap water microorganisms. Additionally, 500 µL of sterile TSB was added to the cup as a source of nutrients for microorganisms to increase their growth rate.

The cup was placed in an orbital-shaker incubator (ES-20, Biosan, Riga, Latvia) at a temperature of 37 °C and shaken at 250 rpm for 24 h. The next day, the membrane samples were stained with fluorescent dyes and the bacterial metabolic activity of potential biofilm was evaluated under a microscope using the slightly modified technique described by Mezule et al. [60]. For that, membranes were briefly rinsed and immersed in a 1.5 mL microtube containing 4 mM 5-cyano-2,3-ditolyltetrazolium chloride (CTC, Sigma-Aldrich, Burlington, MA, USA). The samples were incubated in an orbital-shaker at 37 °C and 150 rpm for 1 h. Then, the samples were rinsed with DI water and immersed in a microtube containing 3–4% formaldehyde for 15 min. The samples were rinsed again and immersed in a microtube with 10 µg/mL 4′,6-diamidino-2-phenylindole (DAPI, AppliChem, Darmstadt, Germany) for 15 min. The stained samples were rinsed again and observed with an epi-fluorescence microscope (Ex: 340/380 nm; Em: >425 nm for DAPI and Ex: 545 ± 30 nm; Em: 610 ± 75 nm for CTC). Here, separate channels were used for each dye to observe only the DAPI-stained sample or only cells containing red insoluble formazan crystals. Image acquisition of each channel was performed using ZEN 3.2 software (blue edition) © (Carl Zeiss, Oberkochen, Germany). Images were combined using the Image Calculator function.

## 4. Conclusions

This study demonstrated the significant impact of additives, such as PVP and ZnO, on the performance of UF flat-sheet membranes. Membranes modified with ZnO exhibited remarkable improvements in the rejection capabilities, albeit with a trade-off in permeability. Modified membranes showed improvements in their wettability properties and surface homogeneity, which led to a decrease in dynamic contact angle hysteresis at a maximum of 19° for the dual-layer ZnO membrane with PVP.

The modified ZnO membranes exhibited antibacterial efficacy, inactivating >99.9% of *E. coli* via photocatalysis or >99% under dark conditions. This offers a promising solution to combat biofouling in such systems. The distinction in efficacy between irradiated and non-irradiated membranes highlights the photocatalytic antibacterial activity of ZnO-modified membranes. Notably, the preservation of antibacterial activity in non-irradiated membranes suggests their potential utility in both photocatalytic membrane reactors and conventional membrane systems. Similar static antibacterial test results in all ZnO membranes, despite obviously different numbers of ZnO NPs on surfaces found in EDX analysis, suggest that the membrane preparation recipe should be explored with various ZnO NP concentrations to identify the optimal ZnO concentration that maintains antibacterial efficacy without compromising membrane permeability. Although dual-layer membranes theoretically offer the advantage of using fewer blended particles, practical implementation revealed higher flux resistance, which resulted in a clean water flux decrease of 38 L/(h·m^2^·bar).

Despite the promising results obtained in static antibacterial tests, it is possible that biofouling mitigation may not be achieved under real filtration conditions. Our study revealed that natural tap water microorganisms can endure and withstand the antibacterial activity of ZnO. Metabolically active microorganisms were present in natural biofilm on the ZnO membranes even after 24 h. Nevertheless, the activity of ZnO may still influence bacterial EPS production, potentially resulting in less stable biofilms. Overall, it prompts a critical inquiry into the utility of tests using laboratory bacterial strains of *E. coli*, the predominant method in antibacterial testing used in such studies. Our research highlights the need for alternative anti-biofouling evaluation tests for membranes.

## Figures and Tables

**Table 1:** Cultivation results of suspension in membrane static antibacterial tests.

Membrane	Log Growth (+)/Reduction (−) after 24 h	
Unmodified (reference; N1)	+4.6 ± 0.1	
	irradiated	Non-irradiated
Single-layer ZnO (Z1)	−3.2 *	−2.6 ± 0.1
Dual-layer ZnO (Z2)	−3.2 *	−2.5 ± 0.4
Dual-layer ZnO with PVP (Z3)	−3.2 *	−2.0 ± 0.3

* Upper detection limit of the method.

**Table 2:** An overview of microbiological tests of similar membranes and their results.

Reference	Membrane Type	Model Organism, Method	Results
This study	PES blended with ZnO NPs	*E. coli* Plate count technique and tap water biofilm fluorescence microscopy	>99.9% inactivated via photocatalysis, >99% inactivated in darkness, No visible impact on tap water organism biofilm formation
Li et al., 2024 [62]	Commercial PES coated with ZnO/PHMB nanocomposite	*E. coli* Plate count technique	Up to 95.23% inactivated (sample 800ZP)
Sosa et al., 2023 [63]	Commercial flat sheet with ZnO deposition using vacuum	*E. coli* and other coliforms, IDEXX Colilert-18	95.9% treated (*E. coli*, sample E5)
Zhan et al., 2022 [30]	Electrospun PAN@OHec nanofiber with ZnO grafting	*E. coli* and *S. aureus* plate count technique	Up to 97.7% inactivated (*E. coli*, sample 6ZnO-g-PAN)
Zhang et al., 2022 [64]	PES blended with rGO/ZnO nanocomposite	*B. subtilis*, *E. coli*, *P. aeruginosa* plate count technique	>90% inactivation
Rajakumaran et al., 2020 [65]	PSU thin-film nanocomposite	*E. coli* Plate count technique	Up to 91 % inactivation (sample ZnO-S/0.03)

**Table 3:** The composition of membranes and their types.

Membrane ^1^	Casting Solution Recipe (wt-% in Solvent)			Casting Thickness (µm)
	PES	ZnO NPs	PVP	
N1	20	-	-	150 ^2^
N2	20	-	1	150 ^2^
Z1	20	5	-	150 ^2^
Z2	20	5	-	100/50 ^3^
Z3	20	5	1	100/50 ^3^

^1^ N1—unmodified membrane; N2—unmodified membrane with PVP; Z1—single-layer ZnO membrane; Z2—dual-layer ZnO membrane; Z3—dual-layer ZnO membrane with PVP; ^2^ Single-layer membrane; ^3^ Dual-layer membrane: the first number represents base layer thickness; the second number represents surface layer thickness.

**Table 4:** Average numerical values of drinking water quality indicators in 2023 [69].

Parameter	Average Value in 2023	Max. Allowable Value
Microorganisms in total	305 CFU/mL	-
Coliform bacteria	0 CFU/100 mL	0 CFU/100 mL
pH	7.3	6.5–9.5
Conductivity	423 µS/cm	2500 µS/cm
Oxidation (KMnO_4_)	2.3 mg/L	5.0 mg/L O_2_

## Data Availability

The research data are available on demand.

## Supplementary Materials

The following supporting information can be downloaded at: https://www.mdpi.com/article/10.3390/molecules29061274/s1, Figure S1: SEM-EDX cross-section images of prepared ZnO membranes at 20 kV, 200 X; Figure S2: Average filtration curves at constant TMP = 1 bar; Figure S3: SEC/GPC results and dextran MWCO determination; Figure S4: Cultivation results of suspension in membrane static antibacterial tests; Figure S5: Pieces of ZnO membranes placed on agar plates after suspension decimal dilution; Figure S6: Surface area measurement of the commercial ZnO NPs used in membrane fabrication; Figure S7: SEM images of the used ZnO nanoparticles at different magnification.
