# Supplementary material for: Photocatalytic Zinc Oxide Nanoparticles in Antibacterial Ultrafiltration Membranes for Biofouling Control
Source: Molecules. 2024 Mar 13;29(6):1274. doi: 10.3390/molecules29061274 (PMC10976060; doi:10.3390/molecules29061274)
Supplement: Supplementary file 1 [file molecules-29-01274-s001.zip › molecules-2871527-supplementary.pdf]

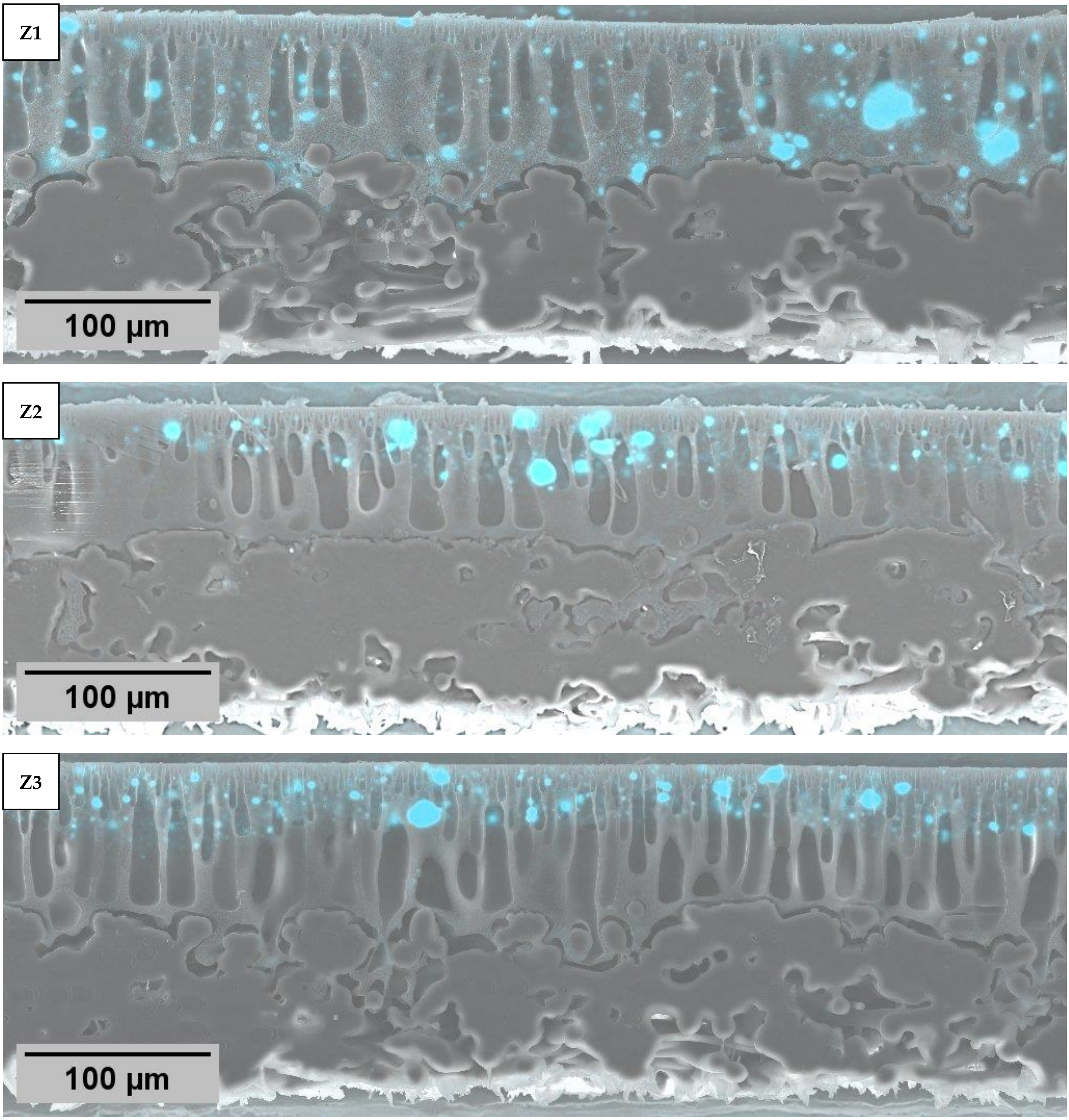

Figure S1. SEM-EDX cross section images of prepared ZnO membranes at 20 kV, 200 X:
Z1 – single-layer ZnO membrane; Z2 – dual-layer ZnO membrane; Z3 – dual-layer ZnO membrane with PVP.

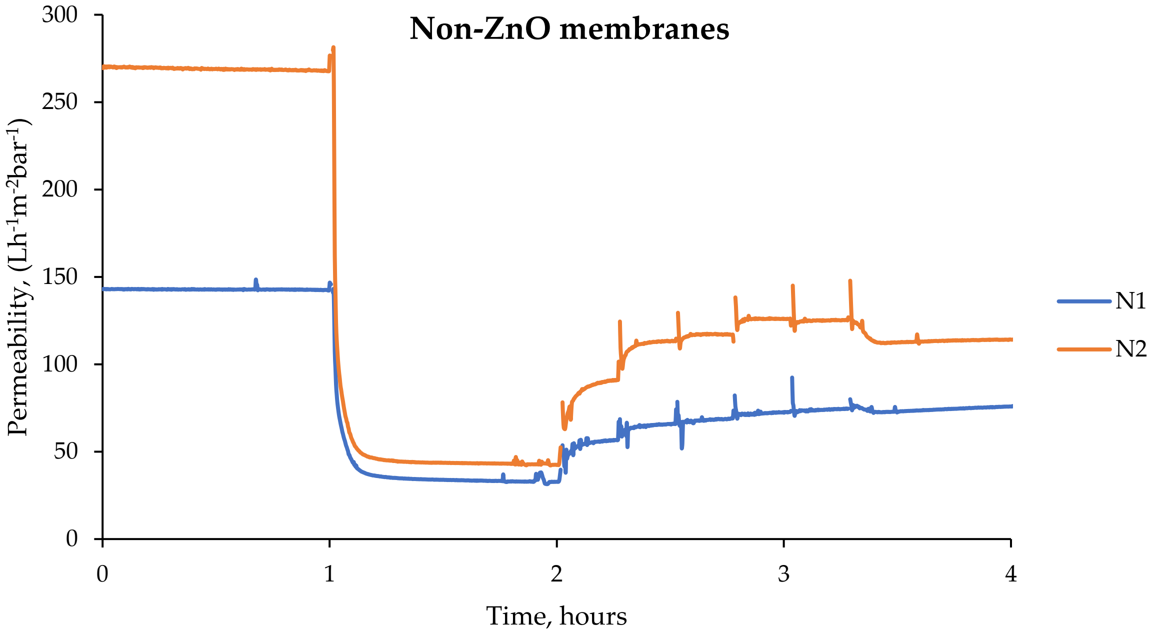

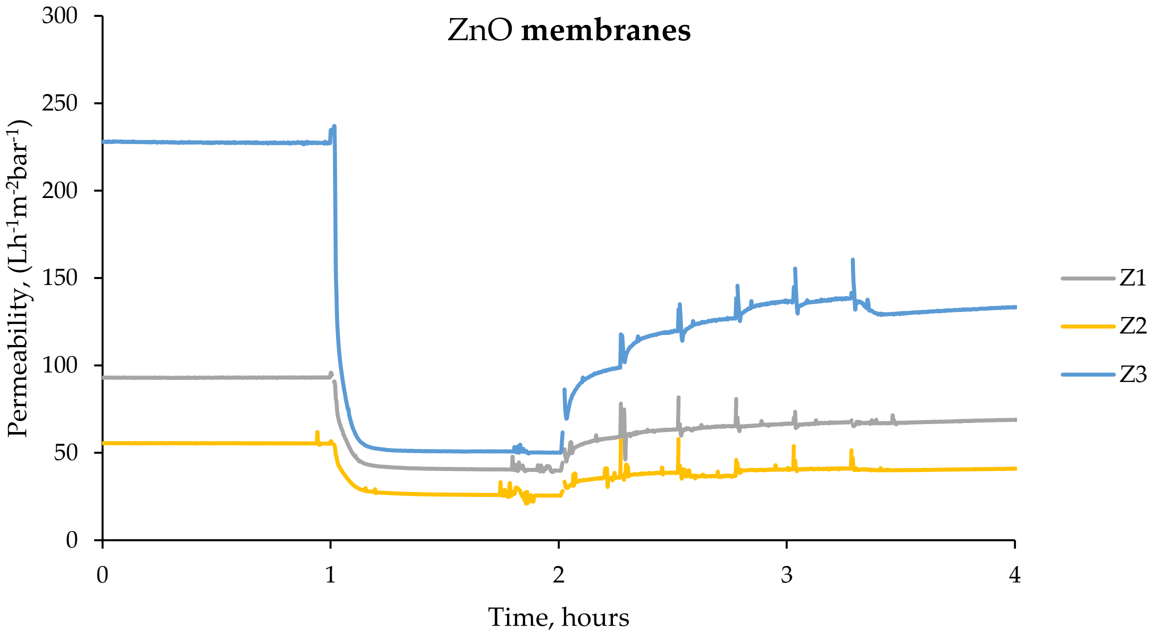

Figure S2. Average filtration curves at constant TMP = 1 bar: N1 – unmodified membrane; N2 – unmodified membrane with PVP; Z1 – single-layer ZnO membrane; Z2 – dual-layer ZnO membrane; Z3 – dual-layer ZnO membrane with PVP.

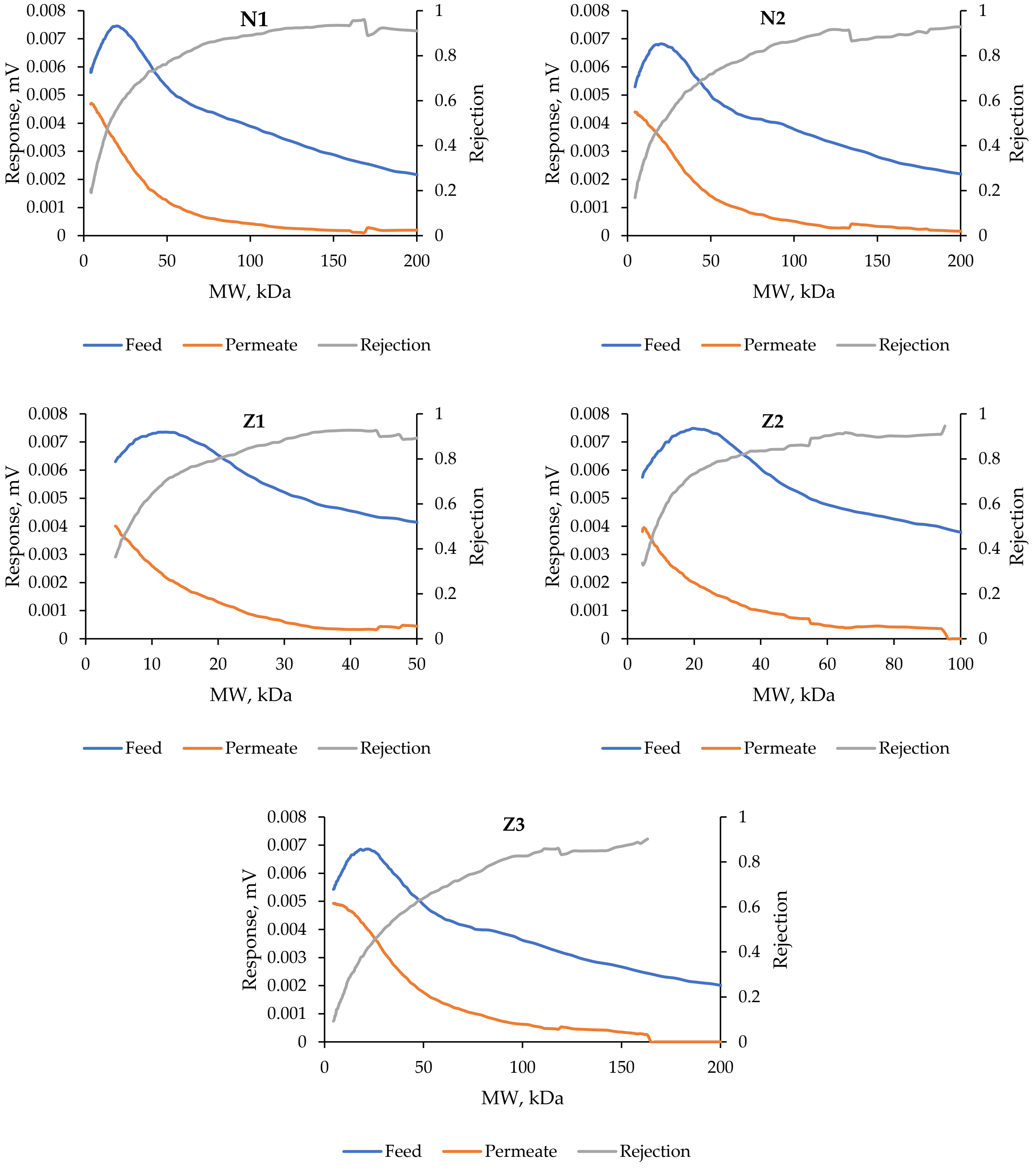

Figure S3. SEC/GPC results and dextran MWCO determination: N1 – unmodified membrane; N2 – unmodified membrane with PVP; Z1 – single-layer ZnO membrane; Z2 – dual-layer ZnO membrane; Z3 – dual-layer ZnO membrane with PVP.

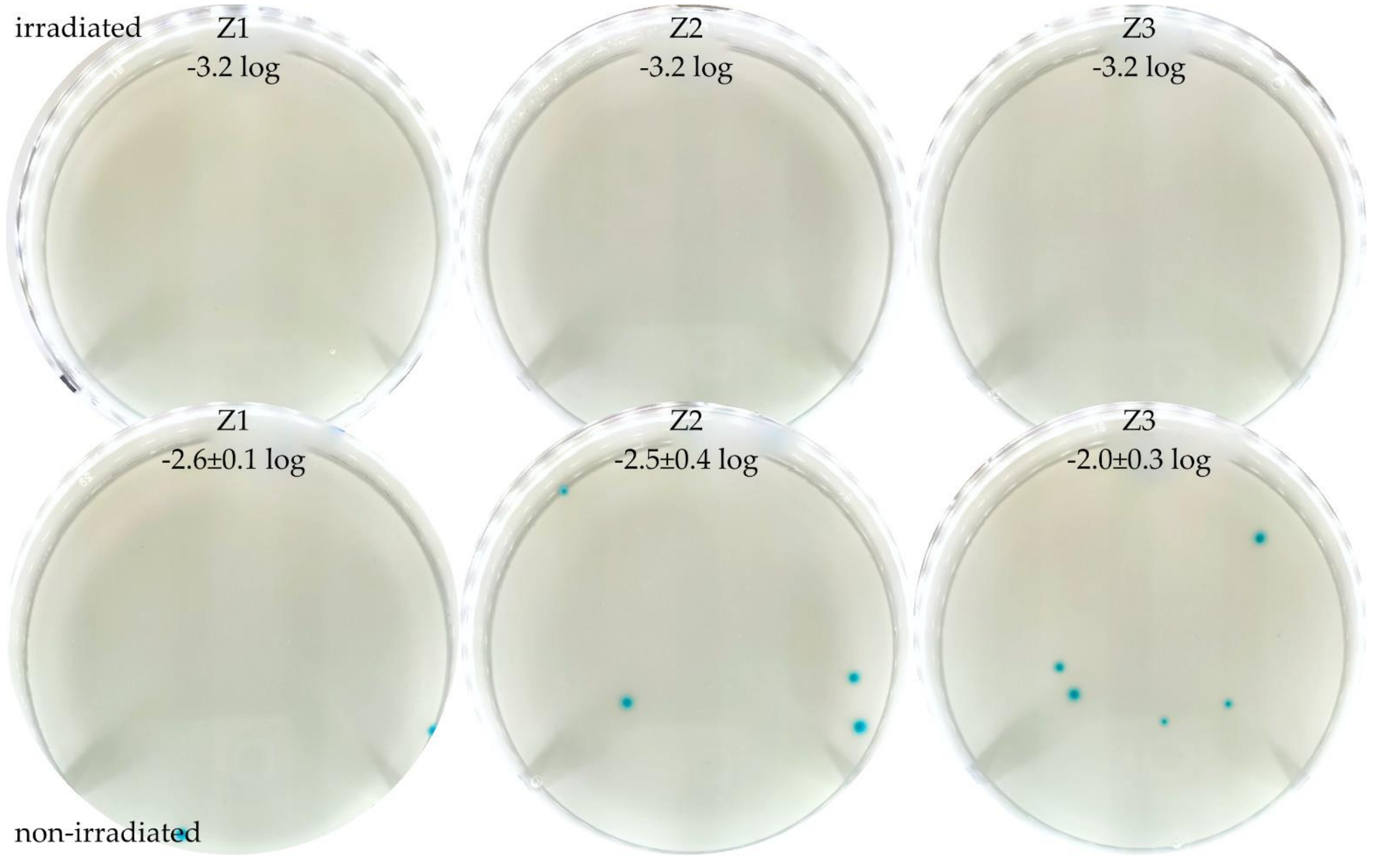

Figure S4. Cultivation results of suspension in membrane static antibacterial tests: Z1 – single-layer ZnO membrane; Z2 – dual-layer ZnO membrane; Z3 – dual-layer ZnO membrane with PVP.

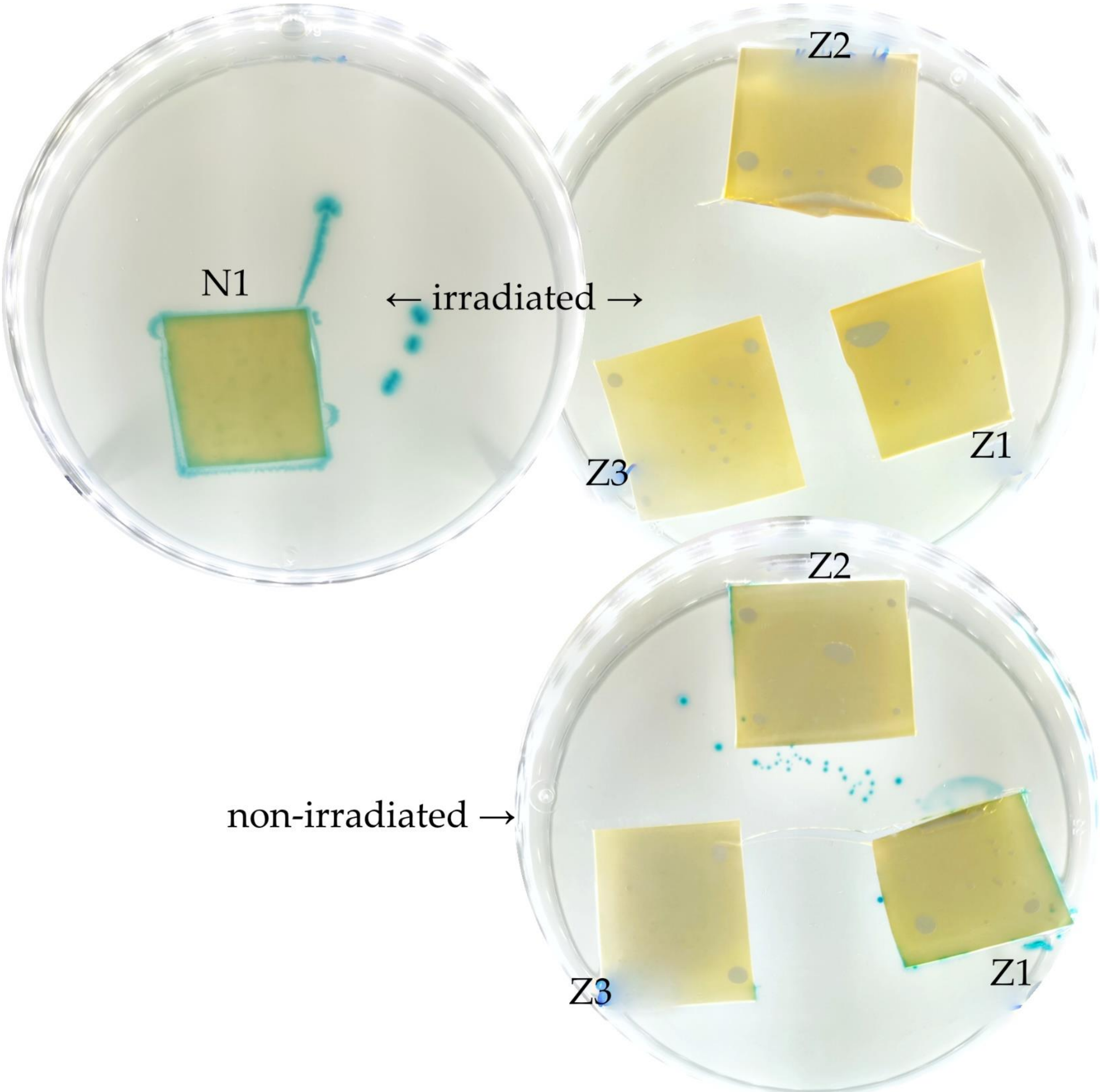

Figure S5. Pieces of ZnO membranes placed on agar plates after suspension decimal dilution: N1 – unmodified membrane; Z1 – single-layer ZnO membrane; Z2 – dual-layer ZnO membrane; Z3 – dual-layer ZnO membrane with PVP.

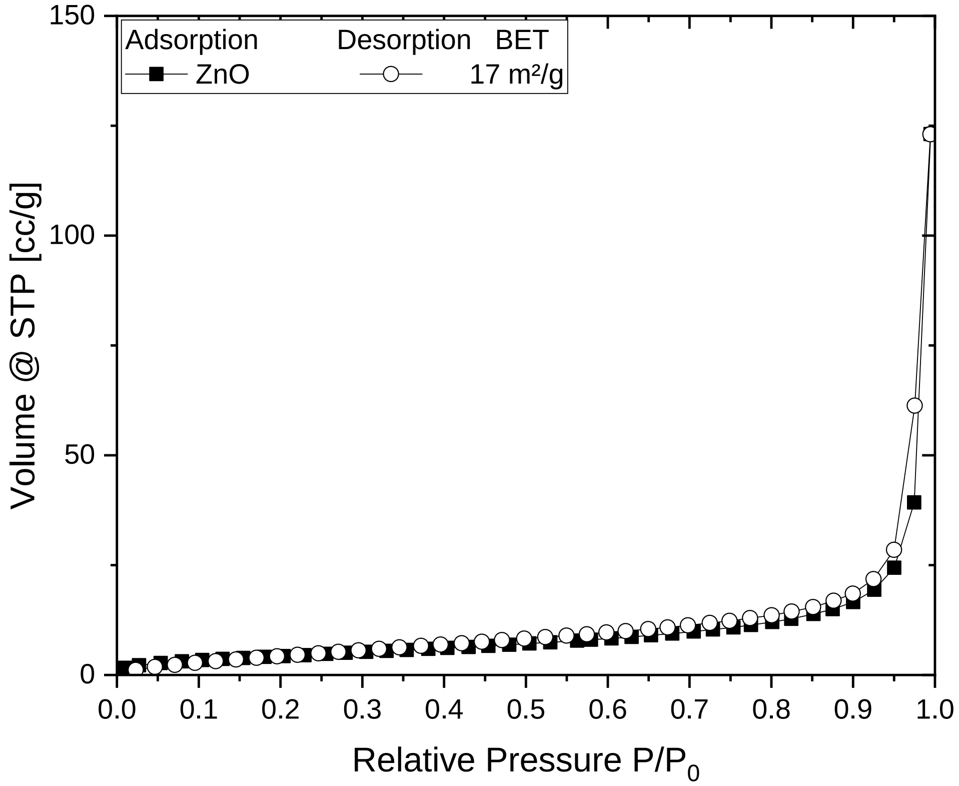

Figure S6. Surface area measurement of the commercial ZnO NPs used in membrane fabrication.

a)

b)

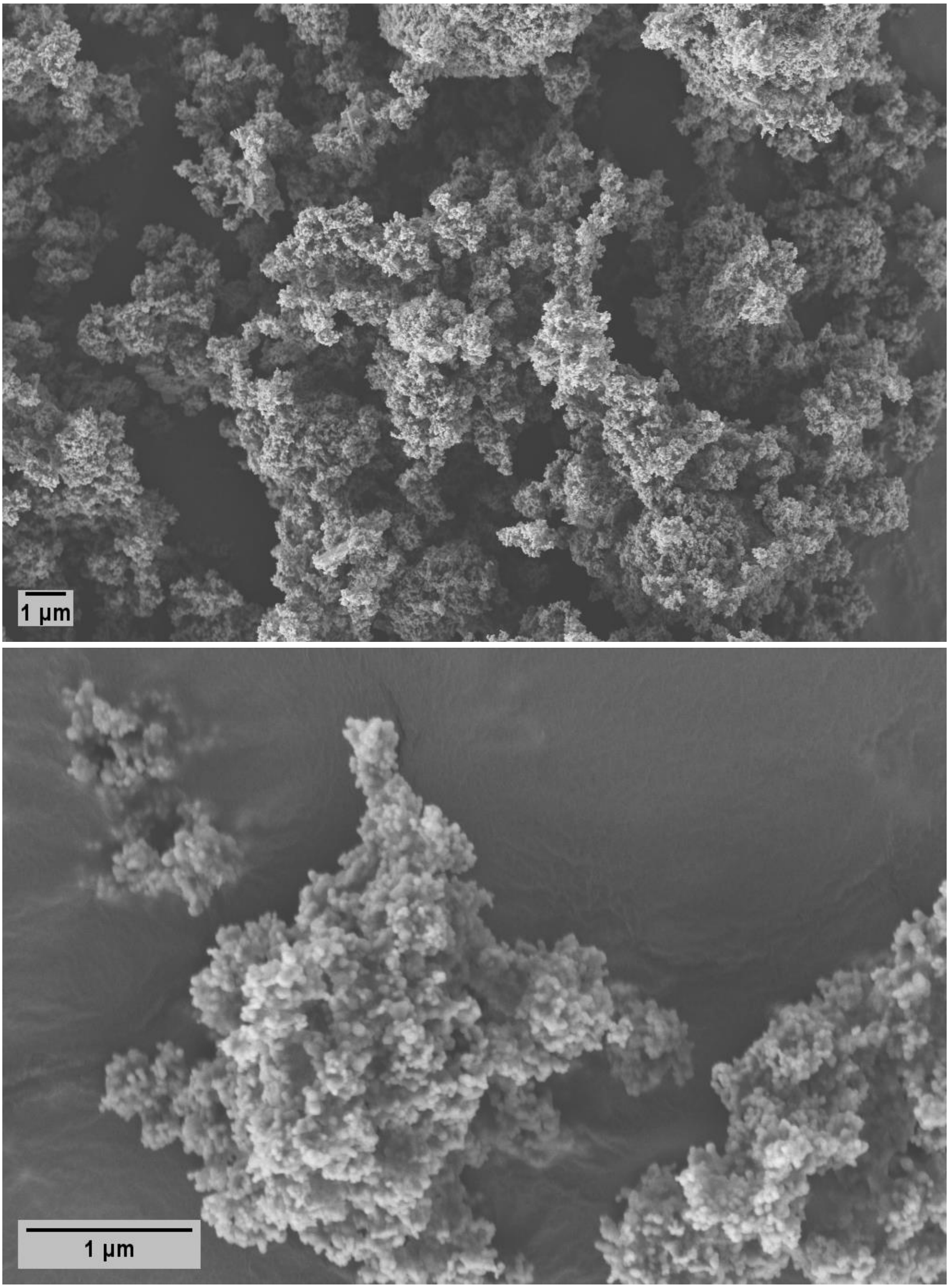

Figure S7. SEM images of the used ZnO nanoparticles at different magnification: 5 kX (**a**); 20 kX (**b**).
